# A Report of a Rare Case of a Solitary Ring-Enhancing Lesion in a Patient With Positive Toxoplasma Serology

**DOI:** 10.7759/cureus.72062

**Published:** 2024-10-21

**Authors:** Brendan P Chernicki, Beatriz Cobo Dominguez, Michael C Huzior, Jessica Caushi, Christopher J Aguirre, Rajiv R Chokshi

**Affiliations:** 1 Internal Medicine, Nova Southeastern University Dr. Kiran C. Patel College of Osteopathic Medicine, Davie, USA; 2 Internal Medicine, Nova Southeastern University Dr. Kiran C. Patel College of Osteopathic Medicine, Clearwater, USA; 3 Internal Medicine, Nova Southeastern University Dr. Kiran C. Patel College of Osteopathic Medicine, Fort Lauderdale, USA; 4 Internal Medicine, Broward Health Medical Center, Fort Lauderdale, USA

**Keywords:** cerebral toxoplasmosis, ring enhancing lesions, seizure, solitary brain lesion, toxoplasmosis gondii

## Abstract

Ring-enhancing lesions are typically observed in patients with immunosuppression caused by underlying conditions such as HIV/AIDS or cancer. These lesions can arise from various etiologies, including infections, neoplasms, or vascular conditions. This case involves a male in his 30s from South America who presented to the emergency department after experiencing a seizure episode. He had no significant prior medical history and reported that he had not seen a doctor since childhood. A comprehensive workup was initiated during his hospitalization, including brain imaging that revealed a solitary ring-enhancing lesion. The evaluation encompassed brain CT and MRI, along with infectious labs measuring inflammatory and serological markers for several diseases. An infectious disease consultant was engaged to assist in developing a thorough assessment and management plan. Throughout the hospitalization, the patient exhibited a benign physical exam with no neurological deficits, remained afebrile, and displayed insignificant white blood cell counts and negative blood cultures. Serological test results were all negative for HIV, rapid plasma reagin, and tuberculosis, except for a positive *Toxoplasma gondii* IgG antibody level. This case is noteworthy because the patient presented with a solitary ring-enhancing lesion in the brain resulting from a prior *T. gondii* infection, despite the absence of documented risk factors for this condition.

## Introduction

Ring-enhancing lesions in the brain are characterized by an area of hypodensity on CT or hypointensity on MRI, surrounded by a rim of enhancing tissue following contrast injection [1]. These lesions can arise from various etiologies, including infectious, vascular, or neoplastic origins. Clinical presentations can vary, often featuring symptoms such as headache, nausea, vomiting, and seizures [1,2]. Differentiating between types of ring-enhancing lesions, such as abscesses and cystic tumors, can be challenging based solely on imaging findings. In these cases, clinical presentation and serological markers play a crucial role in guiding diagnosis. The nonspecific nature of clinical symptoms further complicates the identification of the exact etiology of a lesion, making serological tests even more valuable [3].

Most ring-enhancing lesions are observed in immunocompromised patients and are typically multiple, with *Toxoplasma gondii *being one of the most common causes [4]. *T. gondii *is a protozoan parasite that infects humans through the ingestion of oocysts. These cysts can develop into active forms that are usually contained by the immune system; however, they may breach the blood-brain barrier when immunity is compromised, leading to multiple brain lesions [5]. The seroprevalence of toxoplasmosis in the United States was estimated to be 11.14% between 2011 and 2014, with approximately 6,856 cases reported annually, suggesting that as many as 40 million individuals may be infected nationally [6]. One study indicated that solitary brain lesions in the context of toxoplasmosis were present in 15-30% of patients, with the majority exhibiting multiple scattered lesions [7]. This case presents a patient who was found to have a solitary ring-enhancing lesion, without a history of immunocompromise, and confirmed *T. gondii *infection via serology.

## Case presentation

**Figure 1:**
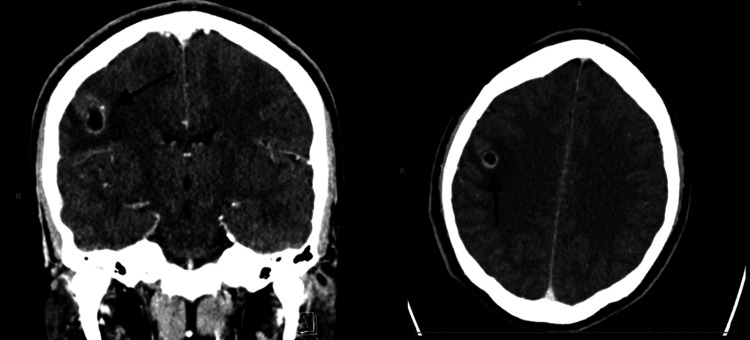
CT scan without contrast showing a solitary brain lesion indicated by the black arrow in both the coronal and axial cuts

A male patient in his 30s from Nicaragua, with no known past medical history, presented to the emergency department after a witnessed seizure. He was sitting on a park bench when he suddenly experienced global left-sided facial weakness and tingling, followed by a brief loss of consciousness. A witness reported that he was shaking for approximately one minute, without any tongue biting or urinary incontinence. Upon waking, the patient appeared confused, prompting the witness to call an ambulance. By the time he arrived at the emergency department, he was alert and oriented. He complained of a mild headache but was otherwise fully neurologically intact. The patient reported that he had moved to the United States a few years prior. A CT scan of the brain without contrast revealed a small cystic and solid enhancing lesion in the right frontal intra-axial region, measuring approximately 1 cm, as shown in Figure 1.

**Figure 2:**
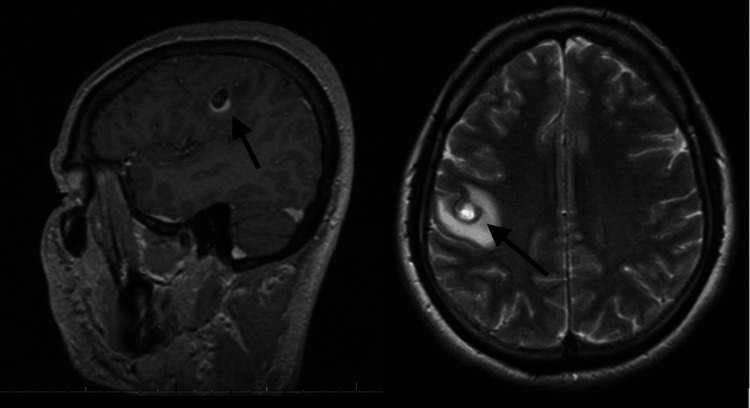
MRI with contrast displaying the ring-enhancing solitary brain lesion, as indicated by the black arrow in both the sagittal and axial cuts

The patient did not complain of dizziness, confusion, nausea, vomiting, headache, weakness, chest pain, or shortness of breath. He was alert and oriented to person, place, and time, and did not exhibit any motor or sensory deficits. He denied a previous history of seizures and stated that he had not seen a physician since childhood. The patient was admitted to the internal medicine team and started on Keppra 500 mg BID and dexamethasone 4 mg every six hours. An MRI with and without contrast was ordered, which revealed a 1.4 cm ring-enhancing lesion within the posterior right frontal lobe, accompanied by central restricted diffusion and associated vasogenic edema. These findings indicated a localized lesion consistent with an inflammatory condition, such as infection, inflammation, or tumor. The MRI images are shown in Figure 2.

The infectious disease service was consulted and suggested that the patient may have neurocysticercosis. He appeared healthy and nontoxic, with a low initial white blood cell count, which only increased mildly after starting steroids. Blood cultures were drawn twice and returned negative at 48 hours. The infectious disease specialist recommended a seven-day regimen of albendazole, along with steroids, pending a normal eye exam. This was later confirmed by the ophthalmology consultant, who found no evidence of ocular or orbital cysticercosis. A full serological workup was ordered, including panels for cysticercosis, TB QuantiFERON Gold, strongyloidiasis, HIV antigen and antibody, and Toxoplasma. Serology results were negative, except for a positive anti-Toxoplasma IgG level greater than 250 IU/mL (reference range for a positive test: >50.0 IU/mL), while IgM was negative. His complete blood count and chemistry labs during the hospital stay are shown in Table 1 and Table 2.

**Table 1:** Complete blood count results during hospital stay

Complete blood count	Day 1	Day 2	Day 3	Day 4	Day 5	Reference range
WBC	10.97	12.22	18.01	18.05	14.23	4.0 × 10^3^/uL-11 × 10^3^/uL
RBC	5.67	5.69	5.5	5.56	5.97	4.3 × 10^6^/uL-5.8 × 10^6^/uL
Hemoglobin	16.5	16.8	16.2	16.2	17.7	13-17.3g/dL
Hematocrit	47.50%	47.90%	46.50%	46.20%	49.90%	38-52%
MCV	83.8	84.2	84.5	83.1	83.6	78-100 fL
Platelets	248	233	234	258	267	140 × 10^3^/uL-400 × 10^3^/uL
Neut %	66.50%	86.50%	87.50%	88.10%	82.70%	37-80%
Lymph %	24.80%	11.90%	8.80%	7.80%	10.60%	20-45%
Neut abs	7.3	10.56	15.76	15.91	11.76	1.8 × 10^3^/uL-7.70 × 10^3^/uL
Diff						
Segs	-	-	91%	-	-	37-80%
Bands	-	-	1%	-	-	0-6%
Lymphs	-	-	7%	-	-	20-45%
Neuts abs calculated	-	-	16.57	-	-	1.8 × 10^3^/uL-7.70 × 10^3^/uL
RBC morph	-	-	Normal	-	-	No reference range

**Table 2:** Chemistry results during hospital stay

Chemistry	Day 1	Day 2	Day 3	Day 4	Day 5	Reference range
Sodium	141	137	137	137	137	136-145 mmol/L
Potassium	4.1 mmol/L	4.2	4.5	4	4	3.5-5.1 mmol/L
Hemolysis	None	None	None	None	1+ (A)	No reference range
Chloride	108	106	107	104	103	98-107 mmol/L
CO2	19	19	19	23	22	22-29 mmol/ L
Anion gap	14	12	11	10	12	5-14
Glucose	126	153	142	127	119	70-105 mg/dL
BUN	10	9	13	18	15	8-21 mg/dL
Creatinine	0.9	0.8	0.8	0.8	0.8	0.8-1.2 mg/dL
GFR	>60	>60	>60	>60	>60	>60 mL/min/1.73m^2^
AST	37	-	21	16	16	5-34 unit/L
ALT	52	-	40	35	35	0-55 unit/L
Prolactin	-	-	-	-	25.9 nanong/mL	3.5-19.4 nanong/mL

The patient’s serology during his hospital stay revealed a positive Toxoplasma IgG antibody level of >250 IU/mL, with the reference range for a positive test being >50 IU/mL. He also had a negative Toxoplasma IgM antibody level of 0.39 IU/mL, where a negative test is represented by values <0.90 IU/mL. Additionally, the patient tested negative for cysticercosis IgG antibodies, had a non-reactive rapid plasma reagin test, and received a negative QuantiFERON tuberculosis test.

The patient was deemed stable for discharge, having shown marked clinical improvement with no new seizure episodes or concerning neurological symptoms. Unfortunately, as he was uninsured and not a United States resident, he was advised to follow up at his local hospital with specialists in infectious disease, neurology, and neurosurgery. He was instructed to return to the emergency room for any seizure-like symptoms or new concerning symptoms, emphasizing the importance of close follow-up with his new provider. The discharge medications included a seven-day course of albendazole and steroids with a taper, as well as a 14-day supply of Keppra.

## Discussion

Ring-enhancing lesions are characterized by a contrast-enhancing halo and can present with a wide variety of clinical symptoms, with differential diagnoses including primary brain tumors, metastases, non-neoplastic cysts, and abscesses [3]. Brain abscesses typically arise from direct local spread of infection or hematogenous seeding from systemic infections. They are commonly associated with fever, headache, nausea, and neurological changes, with fever occurring in 45-50% of patient presentations [8]. In this case, the patient did not exhibit fever or progression of neurological symptoms throughout his hospital course, suggesting that the lesion was likely chronic rather than an acute event. Furthermore, his blood cultures remained negative, and no significant leukocytosis was observed in the lab results. Notably, the patient had a positive *T. gondii *serology IgG test, indicating a past infection, as IgG levels plateau at two to three months post-infection and persist as residual titers for life. The negative IgM serology likely rules out an acute infection [9].

This patient had no known history of immunocompromise, and a negative workup for conditions such as HIV, tuberculosis, or malignancy - recognized causes of solitary, ring-enhancing intracerebral lesions - makes this a unique presentation of *T. gondii *infection. Few cases of immunocompetent patients diagnosed with toxoplasmosis exist, and in those instances, multiple rim-enhancing lesions are typically identified on imaging [10]. The only positive serological finding for this patient was the Toxoplasma IgG antibody, which can persist for years and is a reliable marker for previous infection with the parasite [11]. The uniqueness of this case lies in the fact that, while toxoplasmosis is among the most common parasites causing ring-enhancing lesions, such occurrences are rare in immunocompetent individuals. These lesions are more commonly associated with opportunistic infections in immunocompromised patients, particularly those with AIDS or HIV [12,13]. Additionally, the presence of a solitary ring-enhancing lesion rather than multiple lesions further distinguishes this case from typical presentations associated with toxoplasmosis.

Current literature has documented solitary cerebral lesions linked to a positive Toxoplasma IgG antibody, although, in those studies, patients were often confirmed to have AIDS with CD4 counts as low as 75 cells/mm³. A potential limitation of this case arises from the broad differential diagnoses for ring-enhancing lesions in this patient’s demographic. For example, primary cerebral lymphoma cannot be definitively ruled out; however, the patient’s normal white blood cell count and benign clinical presentation do not strongly suggest this diagnosis, particularly given the absence of mass effect and the presence of central diffusion [10]. Although a brain biopsy could provide clarity on the etiology of the ring-enhancing lesion, it carries risks of hemorrhage, neurological impairment, and even death [14]. Noninvasive diagnostic imaging techniques, such as diffusion-weighted magnetic resonance imaging and MR spectroscopy, could also help differentiate between ring-enhancing lesions and should be considered in further workups [8].

Another limitation of this case is the lack of follow-up regarding long-term treatment, which was affected by the patient’s insurance coverage. Additionally, his potential exposure to *T. gondii *throughout his life remains unknown. The absence of follow-up may have serious implications for this patient’s health, underscoring the importance of ongoing medical care and regular screenings for infectious diseases in underserved populations. The patient was discharged with a prescription to complete his course of albendazole. While pyrimethamine and sulfonamide in combination, or clindamycin as an alternative, are the primary treatments for cerebral toxoplasmosis [15], albendazole was chosen initially due to concerns regarding neurocysticercosis, as this medication effectively penetrates the blood-brain barrier [16].

## Conclusions

Toxoplasmosis is a prevalent parasitic infection that can impact both healthy individuals and those with significant comorbidities, with the latter group experiencing more complications, particularly involving the nervous system. This case presents a unique instance of an individual from South America who had not sought medical care since childhood and was found to have a solitary ring-enhancing lesion alongside confirmed toxoplasmosis serology, with no evidence of other immunosuppressive conditions. Without the seizure episode, this patient might have remained unaware of the brain lesion or the positive toxoplasmosis serology, highlighting the critical importance of seeking medical care throughout life, especially while traveling or living in different countries. Physicians must recognize the unique presentations of conditions such as parasitic infections to provide optimal treatment for disorders that can lead to significant neurological complications.

## References

[1] Chan KY, Siu JCW (2021). Magnetic resonance imaging features of cerebral ring-enhancing lesions with different aetiologies: a pictorial essay. Hong Kong J Radiol.

[2] Tran D, Rahman Q, Weed M, Chow B (2021). Differential diagnosis of a ring-enhancing brain lesion in the setting of metastatic cancer and a mycotic aneurysm. Radiol Case Rep.

[3] Elsadway M, Ali H (2018). Verification of brain ring enhancing lesions by advanced MR techniques. Alexandria J Med.

[4] Garg RK, Paliwal V, Pandey S, Uniyal R, Agrawal KK (2024). The etiological spectrum of multiple ring-enhancing lesions of the brain: a systematic review of published cases and case series. Neurol Sci.

[5] Dian S, Ganiem AR, Ekawardhani S (2023). Cerebral toxoplasmosis in HIV-infected patients: a review. Pathog Glob Health.

[6] Belk K, Connolly MP, Schlesinger L, Ben-Harari RR (2018). Patient and treatment pathways for toxoplasmosis in the United States: data analysis of the Vizient Health Systems Data from 2011 to 2017. Pathog Glob Health.

[7] DiPellegrini G, Boccaletti R, Mingozzi A, Fara A, Policicchio D (2023). Single thalamic localization of brain toxoplasmosis mimicking brain tumors: radiological and clinical findings. Surg Neurol Int.

[8] Bokhari MR, Mesfin FB (2024). Brain abscess. StatPearls [Internet].

[9] Zhang K, Lin G, Han Y, Li J (2016). Serological diagnosis of toxoplasmosis and standardization. Clin Chim Acta.

[10] Basit KA, Nasir S, Vohra E, Shazlee MK (2018). Toxoplasmosis in an immunocompetent patient. Pak J Med Sci.

[11] Mardani-Kataki M, Beiromvand M, Teimoori A, Amari A, Tavalla M (2022). Is immuno-PCR better than ELISA test for detection of Toxoplasma gondii IgG antibody?. Acta Parasitol.

[12] Takayanagui OM, Bonato PS, Dreossi SA, Lanchote VL (2002). Enantioselective distribution of albendazole metabolites in cerebrospinal fluid of patients with neurocysticercosis. Br J Clin Pharmacol.

[13] Okubo Y, Shinozaki M, Yoshizawa S (2010). Diagnosis of systemic toxoplasmosis with HIV infection using DNA extracted from paraffin-embedded tissue for polymerase chain reaction: a case report. J Med Case Rep.

[14] Scott BJ, Douglas VC, Tihan T, Rubenstein JL, Josephson SA (2013). A systematic approach to the diagnosis of suspected central nervous system lymphoma. JAMA Neurol.

[15] Riche M, Amelot A, Peyre M, Capelle L, Carpentier A, Mathon B (2021). Complications after frame-based stereotactic brain biopsy: a systematic review. Neurosurg Rev.

[16] Nath A, Sinai AP (2003). Cerebral toxoplasmosis. Curr Treat Options Neurol.

